# The Premonitory Symptoms of Insanity

**Published:** 1873-12

**Authors:** Horatio R. Bigelow

**Affiliations:** Boston, Mass.


ARTICLE II.— *The Premonitory Symptoms of Insanity.* By HORATIO R. BIGELOW, Boston, Mass.

In his Croonian lectures before the Royal College of Physicians, London,* Dr. Radcliffe alluded to certain conditions of mental habit, which might be considered as precursory of mental alienation. His intelligent observations have been so thoroughly corroborated in certain cases that have fallen under my notice, that I propose to go over this field somewhat more in detail, and to add to it other facts which are worthy of further investigation—especially those relating to the early ophthalmoscopic appearances. The first appreciable symptoms of mental derangement number of the arc do measure my body, it comprehendeth not my mind: whilst I study to find out how I am a microcosm, or little world, I find myself something more than the great. There is surely a piece of divinity in us, something that was before the elements and owes no homage unto the sun." (Religio Medici.)

* Lancet, June and July, 1873.

are invariably announced by one or more of the following conditions:

1. Self-conceit.
2. Misanthropy.
3. Distrust.
4. Impulsiveness.
5. Melancholy.
6. A disordered fancy.
7. Delirious excitement.
8. Disturbances of the senses.
9. Untruthfulness.
10. Moral cowardice.
11. Instability of purpose.

The ocular appearances which I have found co-existing with one or more of the preceding, and which have been followed in due course of time by the actual outbreak of the disease, have been:

1st. Slight anæmia.

2nd. Slight hyperæmia.

3rd. Instability of eye movement, with increased tension (intra-ocular.)

4th. Intermittent irregularity in the supply and distribution of the retinal vessels.

Fresh interest is added to the consideration of these points, from the fact that the nature of the delusion may be generally predicated upon the character of the incipient symptoms, and that the force of the disease may be turned aside, entirely or in part, by a proper attention to the necessities of the case. The symptoms may occur separately, or two or three of the groups may come into conjunction—rarely, if ever, do we find a case in which they are all combined.

It is generally maintained that the ophthalmoscope reveals nothing abnormal in the intra-ocular structures, in the earliest stages of insanity—nothing which may not be a normal variation of the healthy fundus, or a constant symptom of numerous other brain lesions. This, in so far as it relates to anything absolutely pathognomonic, is, in the present infancy of our research, quite true; but I do unhesitatingly affirm, that there are certain conditions of the fundus oculi, which, in connection with the concurring mental symptoms, would justify a diagnosis of approaching alienation. I have noted the one or the other of these conditions in so many cases, all developing into confirmed insanity, that I am persuaded of their very general presence.

As these appearances have not, to my knowledge, been particularly noticed, in this connection, I will take up their consideration at once, and then, should space permit, pass on to a brief review of the mental phenomena.

I. *Slight anæmia*, was observed in ten cases, with the following mental phenomena: in three patients there was misanthropy; in five, great dejection of spirits and melancholy; and in two, suspicions. The alienation generally assumed the form of acute melancholia. The arteries were diminished in size, and the normal proportion of bulk between them and the veins was destroyed; they did not exhibit the same amount of tortuousness as in the normal eye; the discal vessels were with difficulty made out. These symptoms became more prominent as the disease advanced.

II. *Slight hyperæmia.* This condition is characterized by an over supply to the intra-ocular vessels. Presenting this form were fifteen patients—six had delirious excitement; two, instability of purpose; three, melancholy; three, distrust; one, misanthropy. Developing: six, acute melancholia; four, acute mania; one, folie circulaire; four, mania with exaltation. The disc was of a more rosy hue than normal, its vessels slightly enlarged, and new ones, apparently, appearing; the vascular reticulum of the fundus covered a large extent, while the veins were swollen and tortuous. As the disease became established, the above became more and more marked, and finally resulted in an *intense congestion of disc and retina.*

III. *Instability of eye movement, with increased intra-ocular tension.* Three cases were recorded, all characterized by religious fervor, and developing into acute melancholia, which in one case was followed by an attack of maniacal delirium. There was rotary nystagmus in two cases, and horizontal in one. The oscillation was variable, being increased by any nervous shock, or by prolonged reading. There was slight increase of intra-ocular tension in both eyes; slight hyperæmia was visible in the fundus, but as one of the patients was menstruating at the time, it might have been due to that cause. In that case, characterized by horizontal nystagmus, it is supposed that the lesion was due to a commencing cerebro-spinal sclerosis, as some of the other symptoms seem to point to that disease.

IV. *Intermittent irregularity in the supply and distribution of the retinal vessels.* Two cases came within my notice, one with great self-conceit, resulting in melancholia and masturbation, the other in the status epilepticus. The intra-ocular appearances were exceedingly peculiar. At one time the fundus would be quite normal, while its entire character would become changed during the same examination. The retinal vessels, running at first in a natural direction and of a healthy size, would suddenly fade away, to reappear, distended and tortuous; the least excitement was sufficient to induce the change. In neither of the cases could I discover any indications of abnormality in the heart's action, save the usual feebleness of general anæmia.

That some intra-ocular change, more or less evident, must precede every attack of insanity, announcing itself as the first appreciable symptom, seems to me probable. Every lesion of the brain, however obscure in its pathology, must appeal to a disturbed vascular supply for its solution, and the first notice of this change will be given us by the ophthalmoscope. What the chemico-physiological change may be, by which the different forms of insanity are superinduced, is a question as yet beyond our ken, and hence we can apportion to no form an exclusive and particular set of intra-ocular appearances, but from extended observations we can generalize those conditions which are present in certain states of alienation, and by combining these with the mental phenomena, we can predict the result.

It is my belief, that many appearances have been reckoned among the variations of a normal fundus, which are properly the forerunners of commencing disease, and that owing to this latitude of description, many have fallen into the grave error of ignoring slight abnormalities of vision.

The mental phenomena anteceding the disease, will find ample corroboration in the experience of those who have made the study of insanity a specialty. The exaggeration of a vice, or a natural bent of mind, the development of a taste heretofore unrecognized, or the complete change of a virtuous, honest life, into unreliable, unwholesome channels, are indicative of great cerebral disturbance, and demand the closest watching, with the most circumspect treatment. That some of these changes, oftentimes of the slightest appreciable nature, *do* precede every case of alienation, I have no reason to doubt; and looking at mind as the outcome of cerebral action, rather than as a manifestation of an unknown entity—neither matter nor spirit, but possessing somewhat akin to both—this precedence of the disease by the heralds of disturbed function would appear to be eminently probable.

From being an affectionate person, kindly disposed to all, the patient may, at first, manifest notable irritability of temper when reproved, or counseled by parental authority; this gradually grows into dislike of restraint, of association and connection; then, becoming pure misanthropy, it includes the whole family circie: it ignores the closest ties, and even may extend to the human race in general. Combined with this, there is often evinced a suspicious and distrustful disposition, as if they of the household were plotting against the patient's happiness, thus building up a delusion which inevitably results—a delusion or fear of being killed or poisoned.

Not until this evidence has assumed potential form, is the physician consulted, and then the disease must be left to run its course; whereas, the condition of cerebral erythism, which was the essence of the subsequent illusion, might have been dissipated.

In other cases, the person is conscious of a manifest want of *self-control*, of *voluntary power*—he cannot *will* to do that which he desires, and must be *led* like a child to its accomplishment. This want of control, carried to its greatest extent, constitutes *moral insanity.* A singular manifestation of this weakness was once noticed by me, in a patient exhausted by hysteria, and in whom, subsequently, acute mania obtained; a fly alighted on his forehead, when the right arm was raised, almost unconsciously, to drive it away, but ere it was half-way to its journey's end, the question arose in the mind of the patient, whether the left arm would not answer as well—the right arm dropped, and both arms remained passively at the side, while the fly remained undisturbed; the patient, unable to solve that stupendous question of *free will*, could not exert his brain power sufficiently to give the necessary stimulus to either arm.

I have seen patients whose lives had been characterized by stern integrity, unimpeachable honor, and great moral fortitude, suddenly become less watchful in their statements, less circumspect in action, less a "hero in the strife." Little by little, as the cerebral changes received no check, the old self seemed to fade away, and a new creature—unreliable, untruthful, and full of moral cowardice—to obtain sovereignty. Others will assume the most inconsistent and immoral views of the great social laws, sustaining themselves by close logic, and classic reasoning; while others will assume as a reality that which is but the fictitious image of their own imagination. And so on through the series, we meet with cases of confirmed insanity, in all which we may trace the prodromata to some of these changes—often overlooked, so stealthily do they creep on.

In the offspring of neurotic families, and in those where a powerful *neuriasis* is ever calling out strange fancies, or unusual modes of thought, we should especially be on the watch for the least indication—that by healthy association and intelligently directed treatment we may abort the disease.

With Dr. Radcliffe's recently propounded views of mind, I cannot sympathize. Do what I will, I cannot logically enter into that subjective introspection which hypothecates a spiritual entity as presiding over all forms of cerebral action—which from a super-exalted standpoint seeks for a First Cause in the wondrous inter-relationship of a scientifically ordered, intelligible existence—*matter*, with an unknown, unfathomable, suppository ideal—*spirit.* To me the wondrously endowed, chemically organized brain matter, susceptible of infinite variety and change, is a more scientific explanation of mind, the result of these delicate physiological actions, and just as beautiful, as the more vague and intangible, spiritualistic view. Neither in memory, can I recognize anything to controvert this opinion. That a measure of memory exists in the thing remembered, is to me most improbable, as daily evidences occur wherein lapse of time is sufficient to efface entirely, or in part, the integrity of this faculty, by creating great external changes.

